# The Parliamentary Debate on the Cost of National Insurance

**Published:** 1915-07-24

**Authors:** 


### NATIONAL INSURANCE ACT DEVELOPMENTS.

### The Parliamentary Debate on the Cost of National Insurance.

The consideration of the Civil Service Estimates, in so far as they relate to National Health Insurance, in Committee by the House of Commons on Monday, July 12, provided an opportunity, of which there have been too few of late, for reviewing the operations of the National Insurance Acts. The estimates in themselves were not of any outstanding importance. They were published several weeks ago, and we have already commented upon them in our issue dated June 19 (p. 255). They do, indeed, show evidences of the effect of the war by reason of the fact that developments which had been promised, or which might naturally have been expected in normal times, are, in the circumstances, deferred until a more appropriate occasion. The debate was, however, of more than ordinary interest, for it elicited two speeches, from Mr. Charles Roberts and Mr. Handel Booth respectively, which contained matter of first-rate importance.

Mr. Charles Roberts has only within the past few weeks, and since the estimates were framed, been placed in charge of National Health Insurance matters in the House, and it was only natural under these conditions that he should ask the indulgence of members if he should be unable to answer, without notice, questions relating to points of detail in the administration of the Acts. He had, nevertheless, prepared a speech which clearly showed that he has already appreciated the vastness of the problems connected with National Health Insurance, and as Chairman of the Joint Committee it will fall to his lot to undertake the solution of these problems as they present themselves.

Mr. Handel Booth, on the other hand, although not officially connected with the Insurance Commissioners, has probably given greater attention to National Insurance problems than any other member of the House, and in consequence of his knowledge of detail, and of his present independent position, he was able to make suggestions which, if acted upon, should operate to the advantage, not only of the insured population, but also of the State.

### War Help Rendered by the Commissioners.

The first part of the speech delivered by Mr. Roberts was devoted to an interesting *résumé* of the effect of the war on the work of his Department and of the assistance which the Insurance Commissioners have, in various ways, rendered in the conduct of the war. He was able to announce, for instance, that no fewer than 533 clerks had been released from their clerical duties in order that they might undertake military service. Other Government Departments had also received assistance, and, although he did not mention it, we happen to know that very valuable work has been done by the Commissioners in connection with the sale of War Loan vouchers through the agency of Friendly Societies and similar organisations.

He also referred to the effect which the war must inevitably have on the finances of approved societies, though the time is not yet ripe for the full appreciation of the ultimate effects that are likely to be produced.

More important still, however, was the announcement that, " in collaboration with the War Office . . . medical attendance for soldiers on furlough, and for soldiers living at home or in billets, had been secured." This was the principal outstanding requirement in the organisation of National Insurance to meet the necessities created by the war which had been omitted, or overlooked, when the special Amendment Acts were under consideration. We had in this Journal urged the vital necessity for the co-operation of the Insurance Commissioners with the War Office in this matter, and it is therefore of particular interest to us that the provision of medical treatment for the soldier, whilst away from his regimental headquarters, should be made on lines similar to those which we had suggested. So far as we are aware at the time of writing, the arrangements for the co-operation between the two Departments have not been publicly announced, but it was stated by Mr. Roberts that they have already been communicated to the Insurance Committees and the panel committees throughout the country. In due course they will doubtless be published, and the earlier the better; but in the meantime it may be presumed that any soldier in need of medical treatment when on furlough, if he was formerly entitled to benefits under the Insurance Acts, would be able to secure such treatment not only from his own panel doctor, but also from any doctor who undertakes the treatment of insured patients.

Other interesting war points related to the free provision of medical treatment for the dependants of insured persons on military service, the insurance of Belgian workmen, the increase in the price of drugs, and the deflection of the Medical Research Committee from the duties for which it was originally designed to service under the War Office in connection with the compilation of medical statistics, statistics of casualties, and so on.

### The Desire for Economy.

The latter portion of Mr. Roberts's speech was devoted to the question of the possibility of effecting economies in the administration of the Acts. The estimates themselves showed a net reduction, as compared with the previous financial year, of rather less than half a million pounds sterling. This reduction was strictly, however, a war economy, for provision had been made in the estimates for the previous year to allow for the institution of medical referees and for nursing grants, and apart from these items an increased cost would have been shown.

It had been hoped by advocates of economy that it would have been possible to elicit a statement of a considerable saving under the heading of administrative expenses in connection with National Health

Insurance. Such critics were, however, doomed to disappointment, for it was announced that, apart from the items already mentioned, it would only be possible to effect minor administrative economies. It was pointed out that a large proportion of the expenses are beyond the control of the Commissioners, the expenses in question relating to the costs of administering the business of the approved societies. Any saving that the societies can make would, under the Acts, go to increase the benefit funds, and thus there would be no return or direct advantage to the Exchequer.

As a specific instance of the minor economies that may be made, it was announced that it was not intended to fill the vacancy which has recently occurred by the resignation of the legal adviser to the Joint Committee—a post which carries a salary of £1,500.

### Is it Necessary to Change the System?

It was left to Mr. Handel Booth to point out the moral of the apparent impossibility to effect any really considerable economy. The debate had shown the desire for curtailing all unnecessary expenditure, but Mr. Booth had to admit that the Insurance Commissioners had very little control in the matter. They are working under a system rigidly governed by Acts of Parliament. Their duties are prescribed, but at the same time their powers are limited.

The Commissioners cannot alter the Insurance system. That could only be done by Act of Parliament. But Mr. Booth appears to indicate that without a radical change in the system any hope of curtailing expenditure is doomed to failure. He criticised the Commissioners somewhat severely for lack of sympathy with the democratic lines on which it was intended that the Acts should be administered—namely, through self-governing societies. The temptation of the Commissioners—particularly the English Commissioners—to adopt an imperious attitude towards the societies has been complained of, and the number and complexity of the regulations issued perhaps show that in en· the regulations issued perhaps show that in en-quirements of the Acts they have relied upon the highly skilled and trained advisers at their office, with small heed to the fact that such regulations as are really necessary should be couched in language easily understood by the secretaries or acting officials of the societies who have to act upon them.

Although he did not actually say so in so many words, it may be inferred from his remarks on the work of the National Commissions, and the supremacy of the Joint Committee, that he is desirous of seeing the institution of one Commission only, instead of four Commissions as at present, and that upon this body there should be representatives of the societies who, by experience of the practical working of organisations for the encouragement of thrift, should be able to advise as to the best methods to be adopted. Mr. Booth went on to criticise the work of the Insurance Committees, or rather to affirm that without a change of system it would be impossible for the Committees effectively to carry out the duties that have been imposed upon them or to restrict their expenditure to the limits that have been laid down. A point suggested for consideration is whether the Committees should be retained, or if the administration would not be more effective if centralised and placed in the hands of the Commissioners themselves.

### The Date of the First Valuation.

Again, he called attention to the requirement of the National Insurance Act in regard to valuation. It was originally expected that the first valuation would be made when the Act had been three years in force, that is to say that the valuations would be taken in hand at once. Owing to the war, however, and to the desire of obtaining three full years' experience of the payment of benefits under the Act, the date of valuation has been postponed, and it is now intimated that the valuation will probably be made as at December 31 next, although, apparently, no definite decision in the matter has yet been reached.

Periodical valuations are absolutely necessary in order that the finances of approved societies may be prevented from drifting into hopeless insolvency, and in view of the fact that, by their own admission, the Government is satisfied that the benefits which are at present being paid to women are too high as compared with the contributions, it would seem that any delay in making the first valuations must tend to increase the amount of the deficiency and to render more difficult the task of rectification.

